# Rural–Urban Disparities in Health Access Factors Over Time: Implications for Cancer Prevention and Health Equity in the Midwest

**DOI:** 10.1089/heq.2021.0068

**Published:** 2022-05-20

**Authors:** Jordan Baker, Hope Krebill, Hanluen Kuo, Ronald C. Chen, Jeffrey A. Thompson, Matthew S. Mayo, Dinesh Pal Mudaranthakam, Lynn Chollet-Hinton

**Affiliations:** ^1^ Department of Biostatistics & Data Science, School of Medicine, University of Kansas Medical Center, Kansas City, Kansas, USA.; ^2^ University of Kansas Cancer Center, University of Kansas Medical Center, Kansas City, Kansas, USA.; ^3^ Masonic Cancer Alliance, Fairway, Kansas, USA.; ^4^ Department of Radiation Oncology, University of Kansas Medical Center, Kansas City, Kansas, USA.

**Keywords:** rural, health equity, population level, socioeconomic factors, cancer

## Abstract

**Purpose:**

Population-level environmental and socioeconomic factors may influence cancer burden within communities, particularly in rural and urban areas that may be differentially impacted by factors related to health care access.

**Methods:**

The University of Kansas (KU) Cancer Center serves a geographically large diverse region with 75% of its 123 counties classified as rural. Using County Health Rankings data and joinpoint regression, we examined trends in four factors related to the socioeconomic environment and health care access from 2009 to 2017 in rural and urban counties across the KU Cancer Center catchment area.

**Findings:**

The adult health uninsurance rate declined significantly in rural and urban counties across the catchment area (rural annual percent change [APC]=−5.96; 95% CI=[−7.71 to −4.17]; urban APC=−5.72; 95% CI=[−8.03 to −3.35]). Childhood poverty significantly decreased in rural counties over time (APC=−2.94; 95% CI=[−4.52 to −1.33]); in contrast, urban childhood poverty rates did not significantly change before 2012 (APC=3.68; 95% CI=[−15.12 to 26.65]), after which rates declined (APC=−5.89; 95% CI=[−10.01 to −1.58]). The number of primary care providers increased slightly but significantly in both rural and urban counties (APC=0.54; 95% CI=[0.28 to 0.80]), although urban counties had more primary care providers than rural areas (76.1 per 100K population vs. 57.1 per 100K population, respectively; *p*=0.009). Unemployment declined significantly faster in urban counties (APC=−10.33; 95% CI=[−12.16 to −8.47]) compared with rural counties (APC=−6.71; 95% CI=[−8.22 to −5.18]) (*p*=0.02).

**Conclusion:**

Our findings reveal potential disparities in systemic factors that may contribute to differences in cancer prevention, care, and survivorship in rural and urban regions.

## Introduction

Rural regions typically have reduced utilization of preventive cancer screening and greater prevalence of cancer-associated lifestyle factors (e.g., obesity, tobacco, and alcohol use) compared with urban areas, contributing to a higher incidence of lung, breast, colorectal, and cervical cancers.^1^ Additionally, rural areas tend to have fewer physicians and reduced access to health care services, including cancer treatment and surgery.^2–4^ These disparities contribute to increasing cancer mortality rates in rural compared with urban areas, particularly for cancers related to potentially modifiable behaviors.^1,5^

Population-level studies of socioeconomic and environmental factors reveal indicators of cancer health disparities that may inform priorities for health policy, cancer-related research, and outreach activities. For cancer centers seeking to improve their catchment areas' health, understanding the impacts of external factors such as unemployment, health insurance, poverty, and availability of health care services is essential for identifying systemic influences that contribute to poorer cancer-related outcomes for the populations they serve.^6–11^

However, ecological studies of population-level trends are complicated by temporal shifts in health legislation and enforcement of regulations, population migration in and out of geographic areas, and community-level differences across localized regions. For example, differences in tobacco-related regulations have been attributed to smoking rates remaining steady in rural areas, while urban areas report declining smoking rates.^12^

Additionally, introduction of the Affordable Care Act (ACA) in 2010 has contributed to a national decline in the proportion of uninsured persons,^7,8^ improving access to preventive health care. When these population-level factors differ between rural and urban counties, disparities in preventive care can be influenced.

The University of Kansas Cancer Center (KU Cancer Center) serves a unique bistate catchment area that includes ∼4.5 million people living across 123 counties, with all 105 (86 rural, 82%) Kansas counties and 18 (7 rural, 39%) counties in western Missouri. Approximately 1.07 million people (23.67%) live in counties designated as rural. In this study, we explored environmental and socioeconomic factors that may influence cancer burden and health care access among rural and urban regions within the KU Cancer Center catchment area.

Using the KU Cancer Center's Organize Prioritize Trends to Inform KUCC Members (OPTIK) database,^13^ a unique data warehouse integrating publicly available population-level health factors across the geographically diverse KU Cancer Center catchment area, we evaluated temporal trends in the percent of adults without health insurance, the primary care provider rate, the unemployment rate, and the childhood poverty rate, as well as age-adjusted cancer mortality rates.

Ultimately, we aimed to identify environmental and socioeconomic factors that may influence the cancer burden and contribute to differences in health care access in rural and urban regions within the KU Cancer Center catchment area.

## Methods

### Data sources and collection

OPTIK is a data warehouse designed to improve the identification and understanding of challenges faced by the KU Cancer Center and ultimately address the needs of its diverse urban and rural catchment area population. OPTIK includes county-level demographic, cancer risk factor, incidence, and mortality data for the 123 Kansas and Missouri counties that compose KU Cancer Center's catchment area. The data sources and data collection methods within OPTIK have been described in detail previously.^13^

Briefly, the Kansas and Missouri County Health Rankings datasets (2010–2020) are included in OPTIK and collect data for many different health factors and outcomes.^14^ In our analysis, we used the County Health Rankings datasets to obtain annual county-level health factor data from 2009 to 2017 for all counties within the KU Cancer Center's catchment area, as well as the county name, state name, Federal Information Processing System (FIPS code), and Rural–Urban Continuum Code (RUCC). This study was determined to be exempt from IRB review at the University of Kansas Medical Center.

The four health factors were defined according to variable coding within the County Health Rankings as follows: the number of primary care providers (per 100,000 population), uninsured adults (the proportion of adults under age 65 reporting not having health insurance), unemployment (the proportion of adults unemployed and seeking work), and childhood poverty (the proportion of children under age 18 in poverty). Rurality was defined as urban (counties with RUCCs 1–3) and rural (counties with RUCCs 4–9).

Additionally, cancer mortality rates for all cancers overall and the top 5 cancer sites (breast, prostate, lung, colorectal, and melanoma of the skin) were examined to assess whether cancer outcomes differed in rural and urban regions. Age-adjusted cancer mortality rates in Kansas were obtained from the annual Death History records within the Bureau of Health Promotion and Public Health Informatics at the Kansas Department of Health and Environment for years 2008–2012 and 2013–2017.

Given that the rates were available as summary rates aggregated across all rural and urban Kansas counties, statistically significant differences were conservatively identified when 95% confidence intervals for rural and urban estimates did not overlap.

### Statistical analyses

Joinpoint regression [version 4.7.0.0]^15^ was used to analyze temporal trends in all health factors from 2009 to 2017, overall and stratified by rurality (rural vs. urban). All joinpoint models generated annual percent change (APC) estimates and tested the inclusion of up to two joinpoints, representing an inflection point in the estimated slope, for each trend, with a null hypothesis of no joinpoints in any direction across the time period.

A summary average APC estimate was calculated for models with statistically significant joinpoints, representing a weighted average of the trend intervals across the full study period. Models were stratified by rurality and included a test of parallelism that tests a null hypothesis that the regression mean functions estimated for rural and urban counties are parallel, meaning the APC estimates are the same across rural and urban areas over the period. Repeated measures correlation was used to analyze the correlation between all health factors.

Due to missing data, trends for the primary care provider rate were estimated from 2011 to 2017, unemployment from 2009 to 2017, uninsured from 2009 to 2017, and childhood poverty from 2010 to 2017. Statistical significance for all analyses was defined at α=0.05.

## Results

From 2009 to 2017, some socioeconomic and health environmental factors showed similar prevalence according to rurality across the KU Cancer Center catchment area (Table 1). Overall, counties averaged 72.1 primary care physicians per 100,000 population, although rural areas (57.1) showed a lower rate than urban areas (71.6) (*p*=0.009).

**Table 1.** Rural–Urban Differences in the Average Prevalence of Health Equity Factors from 2009 to 2017 in the University of Kansas Cancer Center Catchment Area

	All counties, *N*=123 (SD), Median (range)	Rural counties, *N*=93 (SD), Median (range)	Urban counties, *N*=30 (SD), Median (range)	*p*
Primary care providers^a^	72.1 (30.8)	57.1 (31.8)	76.9 (26.8)	0.009
	53.4 (0–222.5)	53.4 (0–222.5)	53.7 (6.1–124.1)	
Uninsured^b^	13.2 (3.8)	14.4 (3.8)	12.9 (3.3)	0.059
	14.3 (6.1–27.8)	14.9 (6.8–27.8)	12.4 (6.1–23.7)	
Unemployment^b^	5.9 (1.9)	5.2 (1.7)	6.1 (2.1)	0.049
	4.4 (1.8–11.7)	4.2 (1.8–11.3)	5.6 (3.0–11.7)	
Childhood poverty^b^	17.9 (4.9)	19.7 (4.6)	17.3 (5.7)	0.067
	17.5 (5.4–39.7)	17.9 (7.0–32.4)	15.4 (5.4–39.7)	

^a^ *N* per 100,000 population.

^b^ Proportion.

SD, standard deviation.

Across all 123 counties, the uninsurance rate averaged 13.2% (95% CI=[5.75 to 20.72]), unemployment rate averaged 5.9% (95% CI=[2.19 to 9.59]), primary care provider rate averaged 72.1 per 100,000 population (95% CI=[11.78 to 132.41]), and childhood poverty rate averaged 17.9% (95% CI=[8.20 to 27.60]).

There was no statistically significant difference between rural and urban counties in the uninsurance rate (*p*=0.059) or childhood poverty rate (*p*=0.067); however, rural counties (5.2%) had a significantly lower unemployment rate than urban counties (6.1%) (*p*=0.049). Rural counties (57.1 per 100K) also had a significantly lower primary care provider rate than urban counties (76.9 per 100K) (*p*=0.009) (Table 2).

**Table 2.** Annual Percent Change Estimates for Health Equity Factors in Rural and Urban Counties Within the University of Kansas Cancer Center Catchment Area, 2009–2017

	Years	Rural, APC (95% CI)	Urban, APC (95% CI)	Combined, AAPC (95% CI)	Test for parallelism
Primary care providers	2011–2017	0.54 (0.28 to 0.80)	0.54 (0.28 to 0.80)		0.079
Uninsured	2009–2017	−5.96 (−7.71 to −4.17)	−5.72 (−8.03 to −3.35)		0.004
Unemployment	2009–2017	−6.71 (−8.22 to −5.18)	−10.33 (−12.16 to −8.47)		0.016
Childhood poverty	2010–2012	−2.94 (−4.52 to −1.33)	3.68 (−15.12 to 26.65)	−3.25 (−7.08 to 0.73)	0.004
	2012–2017	−2.94 (−4.52 to −1.33)	−5.89 (−10.01 to −1.58)		

AAPC, average annual percent change; APC, annual percent change.

The primary care provider rate was not significantly correlated with any other factor (uninsurance *r*=−0.02, 95% CI=[−0.09 to 0.05]; unemployment *r*=−0.03, 95% CI=[−0.09 to 0.04]; and childhood poverty *r*=−0.03, 95% CI=[−0.11 to 0.04]). Uninsurance showed significant positive correlation with both unemployment (*r*=0.65, 95% CI=[0.61 to 0.68]) and childhood poverty (*r*=0.59, 95% CI=[0.55 to 0.64]). Additionally, unemployment was correlated with childhood poverty (*r*=0.41, 95% CI=[0.35 to 0.46]).

**FIG. 1.**
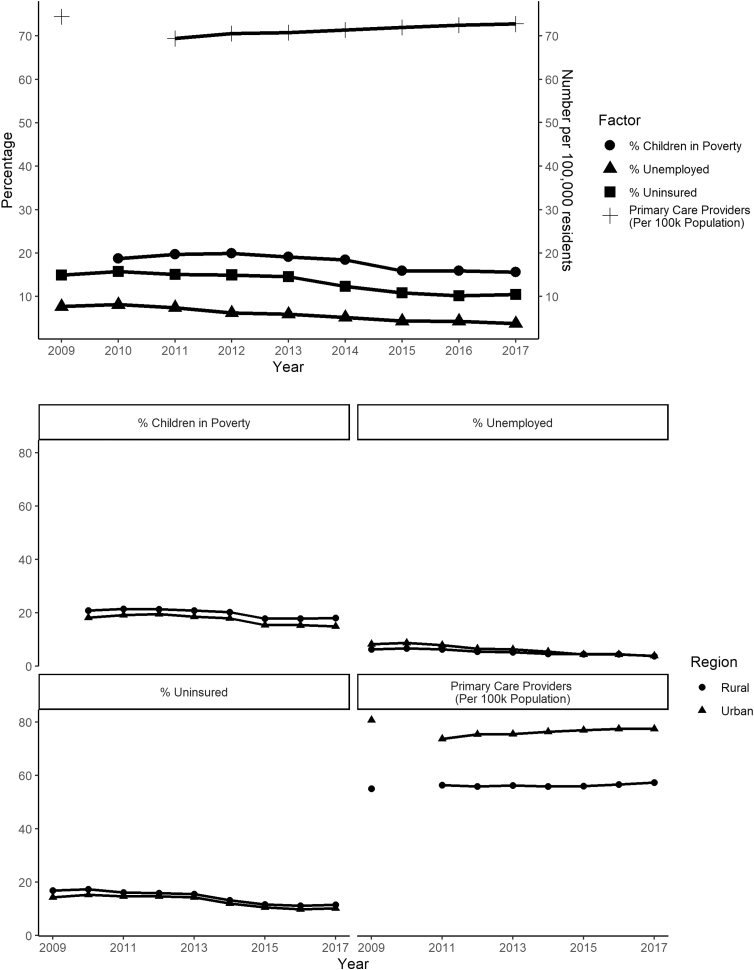
Longitudinal trends in health equity factors in the KU Cancer Center catchment area, overall and by rurality (2009–2017). KU, University of Kansas.

Joinpoint regression models revealed similar temporal trends for the majority of factors within rural and urban areas (Fig. 1). The proportion of adults without health insurance declined over the study period, but at different rates between rural and urban counties (test for parallelism *p*=0.02), with slightly faster decline in rural counties (APC=−5.96; 95% CI=[−7.71 to −4.17]) compared with urban counties (APC=−5.72, 95% CI=[−8.03 to −3.35]).

Regarding childhood poverty, trends were consistently downward for rural counties (APC=−2.94, 95% CI=[−4.52 to −1.33]). A nonlinear trend was observed in urban counties, with rates hovering at ∼19% until 2012 (APC=3.68, 95% CI=[−15.12 to 26.65]), after which childhood poverty in urban regions showed an annual decline of nearly 6% (APC=−5.89, 95% CI=[−10.01 to −1.58]).

Both rural and urban counties saw slight improvements in the primary care provider rate (APC=0.54, 95% CI=[0.28 to 0.80]). The proportion of unemployed adults significantly decreased over time and converged at 4.4% for both urban and rural counties at the end of the study period; however, unemployment declined significantly faster in urban (APC=−10.33, 95% CI=[−12.16 to −8.47]) compared with rural (APC=−6.71, 95% CI=[−8.22 to −5.18]) counties (test for parallelism *p*=0.02).

When comparing age-adjusted cancer mortality rates in the state of Kansas, we observed some disparities between rural and urban counties (Table 3). Rural counties had significantly higher colorectal cancer mortality rates than urban counties for both time periods (2008–2012: rural=16.8 [95% CI=15.8 to 17.9], urban=14.6 [95% CI=13.8 to 15.4]; 2013–2017: rural=16.9 [95% CI=15.8, 18.0], urban=13.5 [95% CI=12.8 to 14.2]).

**Table 3.** Cancer Mortality Rates for the State of Kansas, 2008–2017

	Rural counties, *N*=86, 2008–2012	Urban counties, *N*=19, 2008–2012	Rural counties, *N*=86, 2013–2017	Urban counties, *N*=19, 2013–2017
Overall	173.2 (169.9 to 176.6)	168.4 (165.8 to 171.1)	168.9 (165.6 to 172.3)	156.4 (154.0 to 158.9)
Breast	22.0 (20.4 to 23.8)	20.6 (19.4 to 21.9)	18.9 (17.4 to 20.5)	19.4 (18.3 to 20.7)
Lung	48.6 (46.9 to 50.4)	49.3 (47.9 to 50.8)	45.3 (43.6 to 47.0)	41.2 (40.0 to 42.5)
Colorectal	16.8 (15.8 to 17.9)	14.6 (13.8 to 15.4)	16.9 (15.8 to 18.0)	13.5 (12.8 to 14.2)
Prostate	20.0 (18.3 to 21.8)	18.5 (17.2 to 20.0)	17.6 (16.1 to 19.3)	19.0 (17.7 to 20.4)
Melanoma	2.9 (2.5 to 3.4)	3.1 (2.7 to 3.5)	3.2 (2.7 to 3.7)	2.5 (2.2 to 2.9)

No significant differences were found for lung, prostate, melanoma, or overall cancer mortality rates for 2008–2012. However, for the later part of the study period of 2013–2017, overall (rural=168.9 [95% CI=165.6 to 172.3], urban=156.4 [95% CI=154.0 to 158.9]) and lung (rural=45.3 [95% CI=43.6 to 47.0], urban=41.2 [95% CI=40.0 to 42.5]) cancer mortality rates were significantly higher in rural counties than in urban counties. Breast cancer mortality did not significantly differ by rurality for either time period.

## Discussion

Our county-level socioeconomic and environmental trend analysis reveals significant temporal shifts occurring in the KU Cancer Center catchment area from 2009 to 2017. We observed fewer primary care providers and higher rates of adult uninsurance, unemployment, and child poverty in rural compared with urban regions, demonstrating disparities in key systemic factors related to health equity in cancer prevention and care.^3,9,16,17^

Additionally, we report diverging temporal trends for childhood poverty, uninsurance, and unemployment in rural and urban regions, suggesting that health care access and utilization may disproportionately impact rural areas over time.

We observed that urban counties in the KU Cancer Center catchment area had nearly 20 more primary care providers per 100,000 population than rural areas, with disparities remaining over time. Increases in primary care physician availability did not significantly differ between rural and urban counties. Our findings support a previous report that the national primary care provider rate fell from 2005 to 2015, with greater losses in rural areas.^10^

Lower primary care physician rates have been associated with higher overall mortality rates and higher lung and breast cancer mortality.^2,10,18^ Our results suggest that the availability of primary care physicians may contribute to disparities in cancer care access between rural and urban counties.

We observed that unemployment was higher in urban counties at the start of our study period, with a rapid decline in unemployment across the entire KU Cancer Center catchment area until 2016, when unemployment converged slightly below the national average for both urban and rural counties. Previous studies have reported smaller shifts in unemployment rates within rural counties during and after economic recessions than in urban areas, suggesting greater work stability levels in rural regions.^19^

Our results are consistent with this finding and suggest more rapid job growth in urban areas during our study period. Unemployment has previously been associated with lower cancer incidence rates and higher mortality, particularly for more treatable cancers for up to 5 years, as unemployed persons are less likely to seek medical care.^9,20^

The marked decline in unemployment from 2009 to 2017 across the KU Cancer Center catchment area suggests an environment for increased cancer-related health care and/or incidence trends that may be more pronounced in urban areas.

Our findings reveal temporal shifts in the rate of uninsured adults, which reflect increased availability of health insurance, particularly after implementation of the ACA. We observed higher uninsurance rates in rural areas than in urban areas, which may indicate increased health insurance costs and/or reduced insurance plan coverage or availability in rural regions.^21^

While rural regions did see somewhat greater improvements in uninsurance rates, they still had higher rates at the end of the study period. The introduction of the ACA in 2010 resulted in a national increase in health insurance coverage,^22,23^ which agrees with our findings.

While Kansas and Missouri did not participate in the ACA Medicaid expansion, we found that uninsurance rates declined throughout the period. Our work suggests that access to health insurance for rural and urban areas improved after implementing the ACA in nonexpansion states. Higher uninsurance rates have been associated with later stage cancer diagnosis and increased mortality, and reduced uninsurance rates may reveal future improvements to cancer outcomes within our catchment area.^24^

Similarly, childhood poverty decreased in rural regions throughout the study period while showing no significant change in urban regions until 2012, after which the childhood poverty rate decreased through the end of our study period. We observed slightly increased childhood poverty in rural compared with urban regions, consistent with previous work reporting higher rates of childhood poverty in rural areas with rural children receiving less preventative medical care.^20^

Childhood poverty may measure neighborhood poverty or family socioeconomic status, which has been associated with higher cancer mortality rates.^25^ The declining rates of childhood poverty, health uninsurance, and unemployment within the KU Cancer Center catchment area may reflect an improving socioeconomic environment for more sustainable cancer-related prevention, care, and survivorship efforts in urban areas of Kansas and western Missouri, which exhibit more rapid changes over time.

Increased cancer mortality rates were observed in rural KS counties from 2008 to 2017 for all cancers overall as well as colorectal and lung cancers compared with urban counties, while mortality for other cancer sites was similar across rurality. These results suggest some evidence for disparities in cancer outcomes for rural regions in KS that parallel our findings for the health equity factors.

Specifically, rural regions with higher cancer mortality rates were also where lower primary care provider rates and higher uninsurance rates were observed, revealing an environment of reduced access to health care that may be associated with poorer cancer outcomes in rural areas. These findings are not limited to KS; national studies have also shown higher cancer mortality rates, particularly for lung and colorectal cancers,^1,5,26^ as well as slower declines in mortality over time^1^ among rural versus urban counties across the United States.

Encouragingly, most cancer sites with the highest mortality in KS showed similar mortality rates in rural and urban counties. Higher colorectal and lung cancer mortality in rural KS may reflect a need for targeted outreach and intervention efforts focused on modifiable risk factors and improved health equity for these particular cancers.

Increasing access to cancer care has been a major focus for improving rural health, with numerous studies citing disparities in health insurance, transportation, cancer screening, and the availability of cancer care facilities, providers, and clinical trials as particular challenges to cancer care delivery in rural areas.^27,28^ To better understand rural–urban disparities in cancer outcomes, a recent study highlighted the importance of considering cancer-related environmental and lifestyle factors at the local level.^29^

Our study identified higher health uninsurance rates, higher unemployment, fewer primary care providers, and higher overall, colorectal, and lung cancer mortality in rural counties. Future studies of cancer risk factors, screening, and care delivery within specific rural communities experiencing higher rates of this confluence of health equity factors may improve cancer outcomes for rural patients across the KU Cancer Center catchment area.

Our results should be interpreted in light of some limitations. Our use of County Health Rankings data enabled an evaluation of robust publicly available data comparable with other regions across the United States, although data were not available for some factors throughout our study period. Data were also missing for some rural counties (<4%) due to data suppression for small populations.

Given the ecological nature of our study, we were unable to consider whether the temporal shifts in socioeconomic and environmental factors that we observed may be inter-related or vary across local communities or individuals, and we were unable to examine associations with cancer incidence trends.

However, to our knowledge, our study is the first evaluation of longitudinal trends in systemic factors related to socioeconomic status and health care utilization among rural and urban areas within a large cancer center catchment area, highlighting population-level considerations for cancer prevention and survivorship efforts for communities.

In summary, we identified temporal patterns in rates of uninsured adults, unemployment, childhood poverty, and primary care providers, revealing signs of potential improvement in factors related to socioeconomic status and health care access across the bistate KU Cancer Center catchment area.

Our findings reveal potential disparities in systemic factors related to rurality, which may contribute to differences in cancer prevention, care, and survivorship outcomes in rural and urban regions.

## Abbreviations Used

AAPC: average annual percent change
ACA: Affordable Care Act
APC: annual percent change
FIPS: Federal Information Processing System
KU: University of Kansas
OPTIK: Organize Prioritize Trends to Inform KUCC Members
RUCC: Rural–Urban Continuum Code
SD: standard deviation
